# Prevalence of adjacent segment disease following cervical spine surgery: A PRISMA-compliant systematic review and meta-analysis

**DOI:** 10.1097/MD.0000000000004171

**Published:** 2016-07-08

**Authors:** Lingde Kong, Junming Cao, Linfeng Wang, Yong Shen

**Affiliations:** Department of Orthopedics, The Third Hospital of Hebei Medical University, Shijiazhuang, Hebei, P.R. China.

**Keywords:** adjacent segment disease, cervical surgery, meta-analysis, prevalence, review

## Abstract

**Abstract:**

Prevalence estimates of adjacent segment degeneration (ASD) following cervical spine surgery varied greatly in current studies. We conducted a systematic review and meta-analysis to summarize the point prevalence of ASD after cervical spine surgery.

**Methods:**

Comprehensive electronic searches of PubMed, Embase, Web of Knowledge, and Cochrane Library databases were conducted to identify any study published from initial state to January 2016. Those reporting the prevalence of ASD after cervical surgery were included. A random-effects model was used to estimate the prevalence of radiographic ASD, symptomatic ASD, and reoperation ASD. Univariate meta-regression analyses were conducted to explore the potential associations between prevalence and length of follow-up. All analyses were performed using R version 3.2.3 (R Foundation for Statistical Computing).

**Results:**

A total of 83 studies were included in the meta-analysis. The prevalence of radiographic ASD, symptomatic ASD, and reoperation ASD after cervical surgery was 28.28% (95% confidence interval [CI], 20.96–36.96), 13.34% (95% CI, 11.06–16.00), and 5.78% (95% CI, 4.99–6.69), respectively, in a general analysis. It was found 2.79%, 1.43%, and 0.24% additions per year of follow-up in the incidence of radiographic ASD, symptomatic ASD, and reoperation ASD, respectively.

**Conclusion:**

This meta-analysis provides some details about the prevalence of radiographic ASD, symptomatic ASD, and reoperation ASD after cervical spine surgery. However, the results of this meta-analysis should be interpreted with caution because of the heterogeneity among the studies.

## 1 Introduction

Cervical degenerative disease is a pathological condition that affects the adult spine and is a common cause of cervical radiculopathy and myelopathy in older patients. Cervical surgery can decompress the neural elements and stabilize the affected segments at the same time. However, adjacent segment degeneration (ASD) is often observed in patients who are followed for a long period.^[1,2]^

Postoperative degenerative changes include both radiographic ASD and symptomatic ASD. Radiographic ASD could develop into symptomatic ASD, which correlates with some clinical findings, and symptomatic ASD could lead to serious pain, dysfunction or need for additional surgery. Hilibrand et al^[3]^ reported an incidence of 2.9% per year of the development of symptomatic ASD after single-level anterior cervical discectomy and fusion (ACDF) and estimated that about 25.6% of patients would have symptomatic ASD within 10 years after their index surgery. They also found that more than two-thirds of patients developing symptomatic ASD experienced failure of conservative treatment and required surgical procedures.

A reliable estimate of the prevalence of postoperative ASD is important for informing efforts to prevent, treat, and identify causes of ASD. Over the past few years, several meta-analyses have reported on ASD after lumbar or spinal surgery,^[4–6]^ but to the best of our knowledge, no comprehensive meta-analysis of the epidemiological data on ASD following cervical surgery has been published. Thus, we conducted this systematic review and meta-analysis to obtain accurate figures on the prevalence of ASD after cervical surgery.

## 2 Methods

This study followed the systematic review methodology proposed in the Preferred Reporting Items for Systematic Reviews and Meta-Analyses (PRISMA) statement.^[7]^ As all the analyses were performed by using data extracted from published trials, it is not necessary to obtain ethical approval for this study.

### 2.1 Literature search strategy

We searched PubMed, Embase, Web of Knowledge, and Cochrane Library databases for articles published up to January 2016, using the following search terms: (“adjacent level” or “adjacent segment”) and (“pathology” or “disease”) and (“cervical” or “spine” or “spinal”). The references of all publications were also retrieved to obtain further publications.

### 2.2 Inclusion and exclusion criteria

The following criteria were used for screening the literature. First, the study design included randomized controlled trials, cohort study, case–control study, and cross-sectional study. Second, sample size and point prevalence of ASD were provided or could be calculated. Third, the method of diagnosing ASD was described. Fourth, population was restricted to patients after cervical surgery.

Publications were excluded if they were review articles, case reports, editorials, or letters. Any biomechanical studies or clinical studies investigating cervical tumor, infection, or trauma were also excluded. The number of levels treated with total disc replacement (TDR) or ACDF was not a criterion for exclusion.

### 2.3 Data extraction and outcome measures

For each included study, the following information was extracted: first author, year of publication, country, sample size, surgical approach, study design, length of follow-up, and number of patients with ASD after surgery. The most comprehensive publication was used when several studies involved the same population.

### 2.4 Diagnosis of ASD and other criteria

Three main results were investigated in this study: radiographic ASD, symptomatic ASD, and reoperation ASD. Radiographic ASD was defined as radiographic changes at levels adjacent to a previous surgical segment. Symptomatic ASD is 1 type of radiographic ASD that leads to a new development of radiculopathy or myelopathy. Symptomatic ASD that required a further surgical intervention was considered reoperation ASD.

According to the different length of follow-up, <5 years was considered short-term follow-up; any period longer was considered long-term follow-up.

### 2.5 Assessment of methodological quality

Each included study was chosen independently by 2 authors using a published quality rating system designed especially for articles reporting on prevalence.^[8]^ This 5-point scale system included whether the study design was appropriate for obtaining prevalence estimates; the sample was representative of the general population of patients after cervical surgery; the ASD diagnostic criteria were acceptable; diagnosis of ASD was performed on a consecutive or random sample of subjects; and the final diagnosis was known for 80% of eligible subjects.

### 2.6 Statistical analysis

We extracted data from each study, calculated the overall prevalence of ASD or risk ratios (RRs) with 95% confidence intervals (CIs), and obtained corresponding forest plots. Subgroup analysis was also conducted to discover the prevalence of different categories (number of operated level, approach, and type of surgery). In addition, we restricted analysis to randomized controlled trials that compared TDR and ACDF to investigate the different prevalence of reoperation ASD better between fusion and nonfusion techniques. Furthermore, univariate meta-regression analyses were conducted to explore the potential associations between prevalence and length of follow-up. Heterogeneity among studies was assessed by I^2^ and Q tests. If I^2^ value was <50% and *P* value was >0.10, it was considered significant heterogeneity. In this study, random-effects model was used to pool the results. The influence of individual studies on the overall prevalence estimate was explored by serially excluding each study in a sensitivity analysis. Begg test was used to test the publication bias.

All analyses were performed using R version 3.2.3 (R Foundation for Statistical Computing). *P* value of <0.05 was considered statistically significant.

## 3 Results

### 3.1 Literature search results

**Figure 1:**
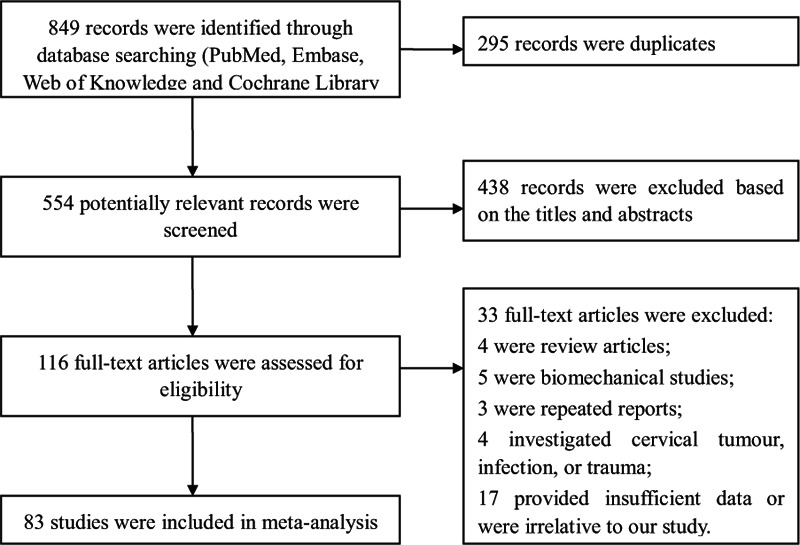
Flow diagram of the study selection process in the meta-analysis.

The first searches give a total of 849 records, and 295 records were duplicates. After review of the titles and abstracts, 438 were excluded. We retrieved full articles for further assessment, and 33 records were further excluded. Finally, 83 studies were included in the meta-analysis.^[1–3,9–88]^Figure 1 shows the details of the screening process.

### 3.2 Study characteristics

Of the 83 studies, 35 reported radiographic ASD, 24 reported symptomatic ASD, and 52 reported reoperation ASD. There were 4050, 4475, and 13,116 patients involved in the 3 types of ASD, respectively. The studies were from 14 countries. Among them, 36 took place in North America, 29 in Asia, 17 in Europe, and 1 in Oceania. The surgical procedures included ACDF, TDR, laminectomy, laminoplasty, posterior foraminotomy, and posterior cervical fusion. The quality score of the included studies ranged from 3 to 5 points. Detailed information on all included studies is shown in Table 1 .

**Table 1:**
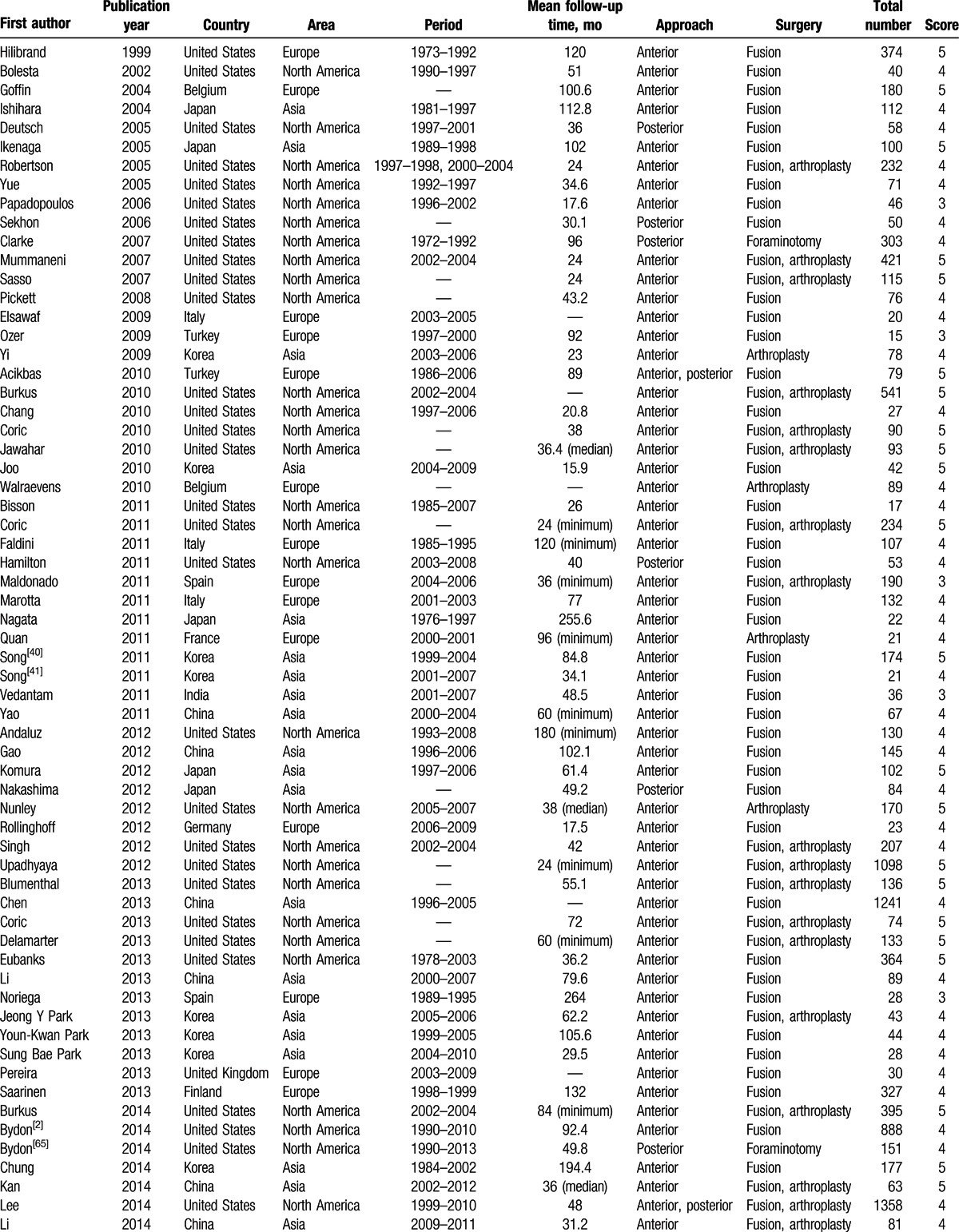
Basic characteristics of included studies.

**Table 1 (Continued):**
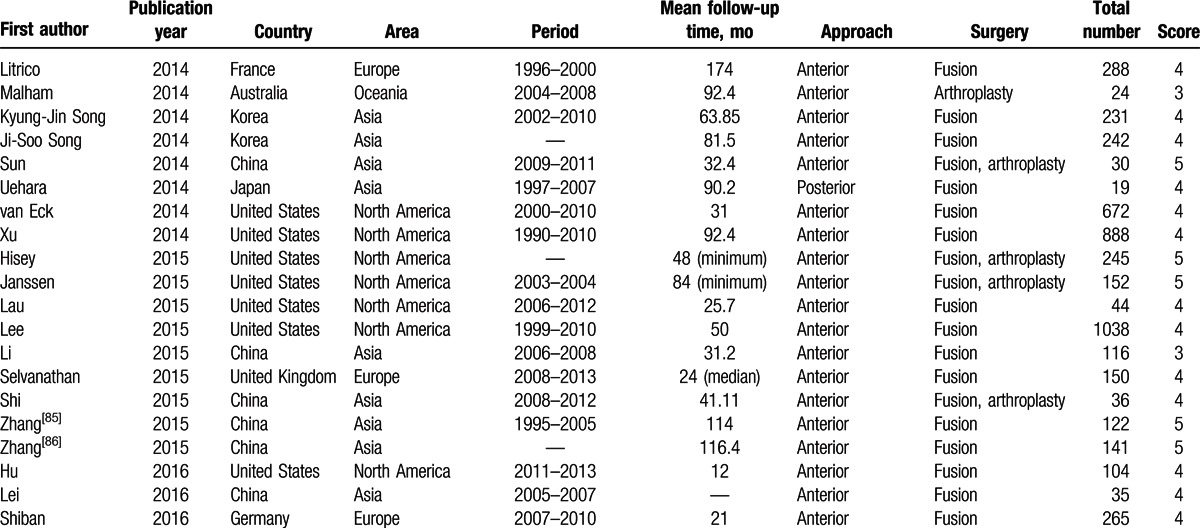
Basic characteristics of included studies.

### 3.3 Prevalence of ASD

**Figure 2:**
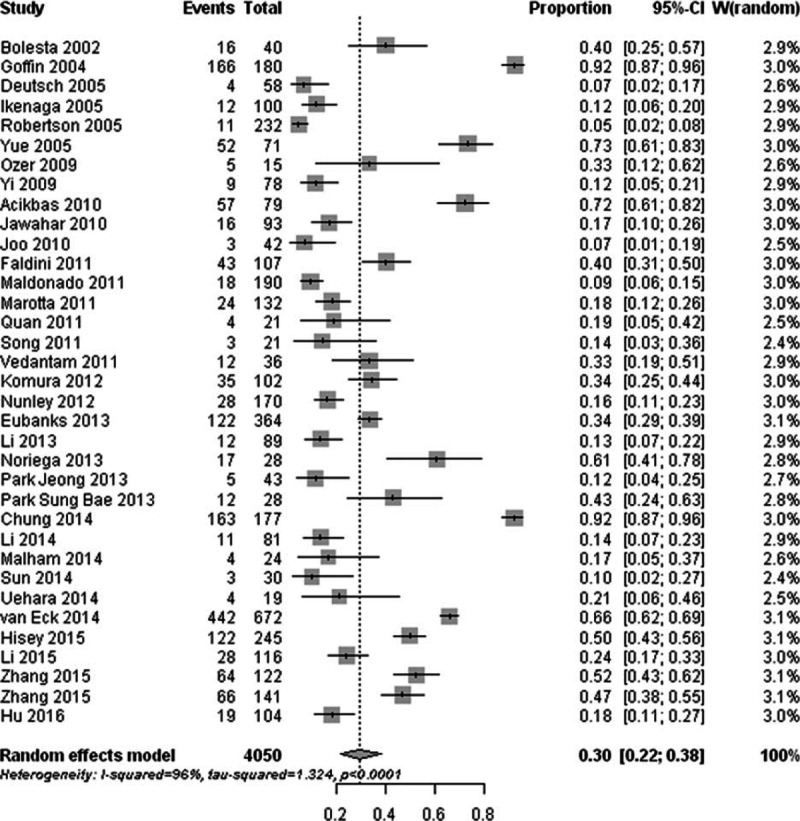
Forest plot of the prevalence of radiographic adjacent segment degeneration after cervical spine surgery.

Thirty-five studies reported the prevalence of radiographic ASD after cervical surgery and revealed that the occurrence of radiographic ASD ranged from 4.74% to 92.22%; the pooled prevalence was 28.28% (95% CI, 20.96–36.96) (Fig. 2). There was significant heterogeneity for radiographic ASD (I^2^ = 95.90%; Q = 837.75; *P* < 0. 01).

**Figure 3:**
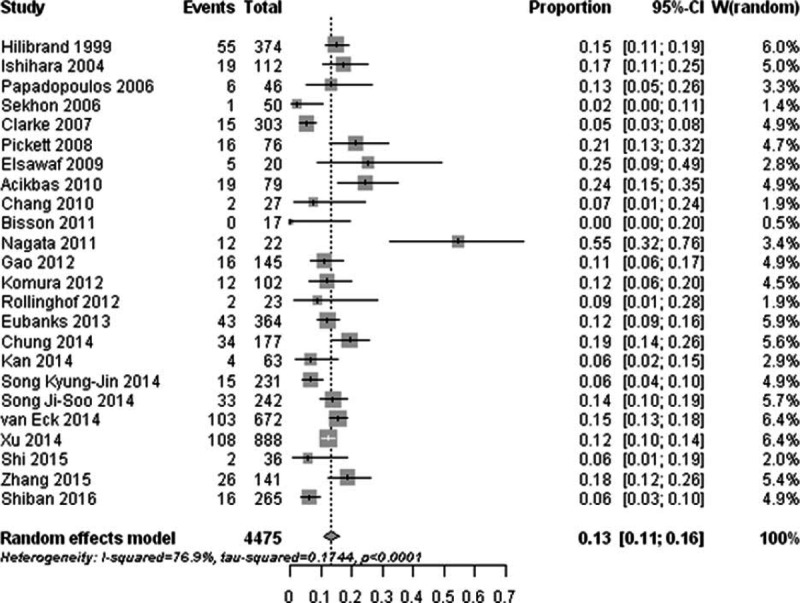
Forest plot of the prevalence of symptomatic adjacent segment degeneration after cervical spine surgery.

The prevalence of symptomatic ASD ranged between 0% and 54.55% in 24 populations. The summary prevalence of symptomatic ASD was 13.34% (95% CI, 11.06–16.00) with significant heterogeneity (I^2^ = 76.90%; Q = 99.67; *P* < 0. 01) (Fig. 3).

**Figure 4:**
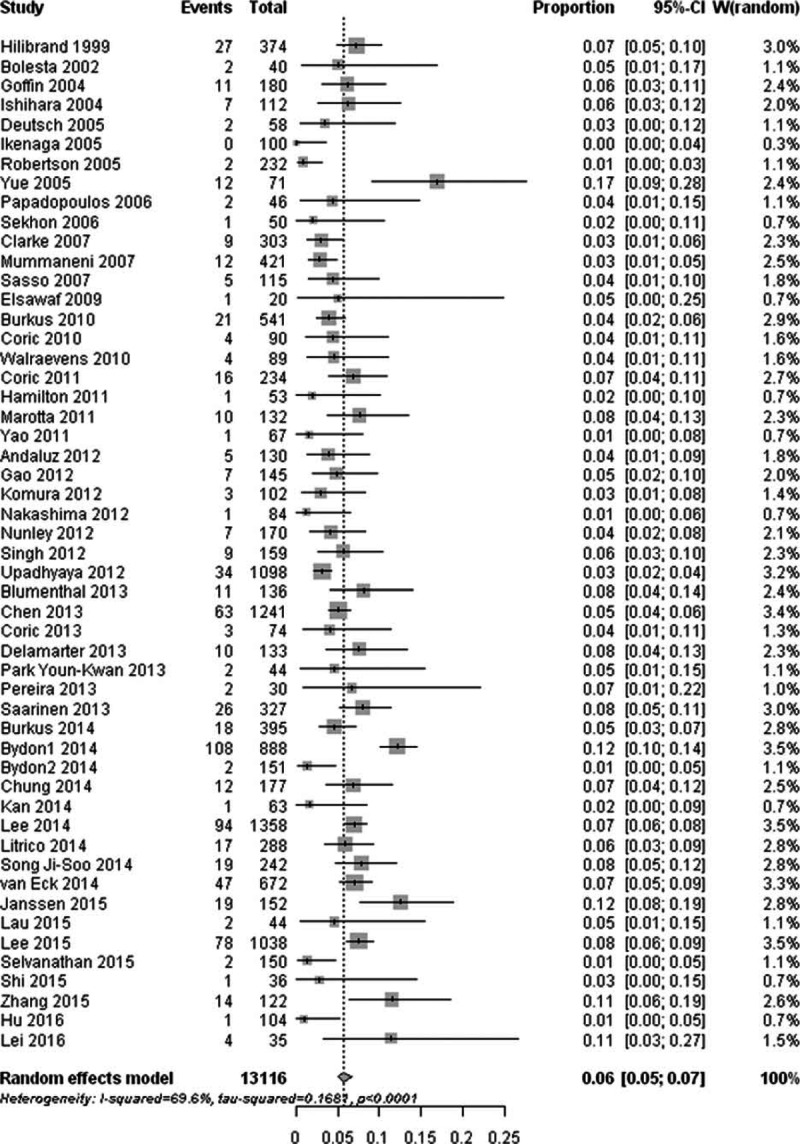
Forest plot of the prevalence of reoperation adjacent segment degeneration after cervical spine surgery.

Reoperation ASD was reported in 52 studies, and the prevalence of reoperation ASD ranged from 0% to 16.90%; the pooled prevalence was 5.78% (95% CI, 4.99–6.69) (Fig. 4). There was significant heterogeneity (I^2^ = 69.60%; Q = 167.58; *P* < 0. 01) for the reoperation ASD.

### 3.4 Prevalence of ASD by study-level characteristics

The studies were divided into short-term subgroup and long-term subgroup according to different length of follow-up, and then we summarized the stratified prevalence estimates based on number of level, approach, and surgery types. The pooled prevalence of ASD after single level, anterior approach and fusion surgery was higher than that after multiple level, posterior approach and arthroplasty. All main results of subgroup analyses are shown in Table 2.

**Table 2:**
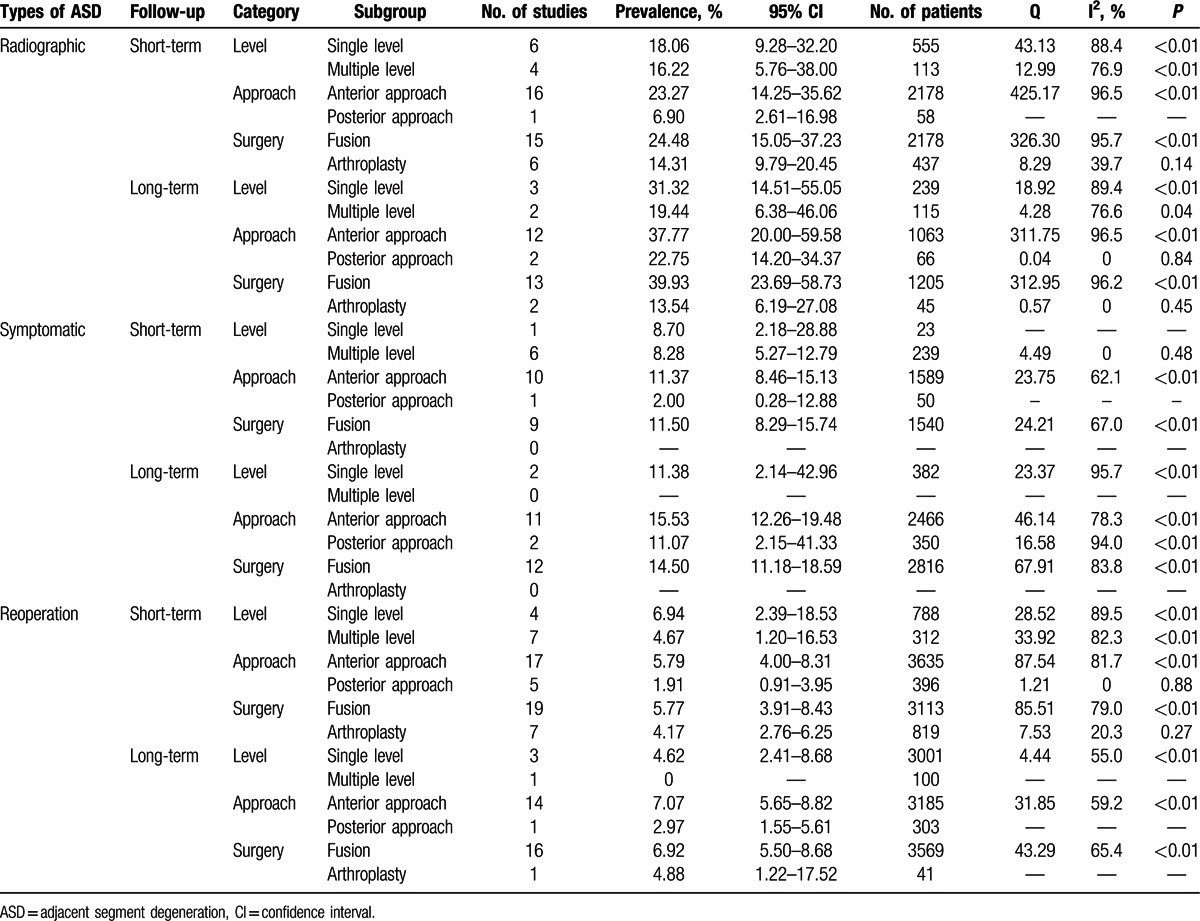
Main results of subgroup analyses.

### 3.5 Comparison of ASD between ACDF and TDR

**Figure 5:**
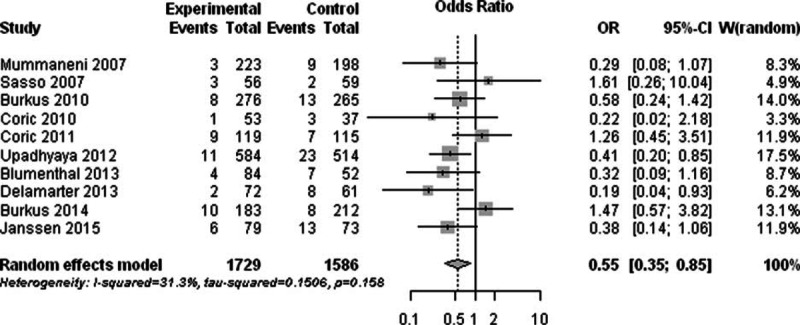
Forest plot showing the risk ratio of reoperation adjacent segment degeneration between the total disc replacement group and the anterior cervical discectomy and fusion group.

Ten high-quality RCTs compared the prevalence of reoperation ASD after 1- or 2-level TDR with ACDF.^[19,20,26,28,33,51,52,55,64,80]^ The test for heterogeneity was not significant (I^2^ = 31.3%; Q = 13.1; *P* = 0.16). The prevalence of reoperation ASD was significantly lower in the TDR group compared with the ACDF group (RR, 0.55; 95% CI, 0.35–0.85; *P* < 0.01) (Fig. 5).

### 3.6 Length of follow-up and prevalence of ASD

Because the longitudinal studies reported different prevalence at different follow-up periods, we also investigated the association between length of follow-up and prevalence of ASD. The results of univariate meta-regression analysis found an addition of 2.79% (*P* < 0.01), 1.43% (*P* < 0.01), and 0.24% (*P* = 0.03) per year of follow-up in the development of radiographic ASD, symptomatic ASD, and reoperation ASD, respectively.

### 3.7 Sensitivity analysis and publication bias

Sensitivity analysis, in which the meta-analyses were serially repeated after exclusion of each study, demonstrated that no individual study affected the overall prevalence estimate of symptomatic ASD or reoperation ASD by more than 1%. Particular individual studies were affecting the overall prevalence estimate of radiographic ASD >1% but <2%.

The funnel plot found an apparent publication bias in the assessment of reoperation ASD (*P* = 0.02). No publication bias was found in the assessment of radiographic ASD (*P* = 0.49) or symptomatic ASD (*P* = 0.49).

## 4 Discussion

The pooled data of this meta-analysis showed that the prevalence of radiographic ASD, symptomatic ASD, and reoperation ASD after cervical surgery was 28.28%, 13.34%, and 5.78%, respectively. It showed that nearly half of the radiographic ASD patients would develop symptomatic ASD, and less than half of the symptomatic ASD would need additional cervical surgery.

The definition of radiographic ASD varied from 1 study to another, but the 1 proposed by Hilibrand et al^[3]^ was used most: they divided ASD into 4 stages (from 1 to 4) according to plain radiography and magnetic resonance imaging, and stage 2 to stage 4 were considered indicative of radiographic ASD. However, in this meta-analysis, the number of studies using this definition was limited; thus, we cannot draw any convincing conclusions based on this definition. The unclear definition is the main cause of significant heterogeneity among studies in the assessment of radiography ASD. We think it could be better to assess and report radiographic ASD by using a unified classification of severity if possible. The definition of reoperation ASD, on the other hand, was more practical because it is much easier to be unified and thus, could provide more robust results and more precise information to us.

Because there was substantial clinical and statistical heterogeneity, subgroup analyses were performed to explore the sources of heterogeneity and clarify the prevalence of different categories. We found that the prevalence of ASD in multilevel subgroup was lower than that in single-level subgroups, and this result was consistent with the study conducted by Hilibrand et al.^[3]^ This finding may be explained by analysis of the levels that underwent surgery. ASD was more likely to occur at the C5/6 and C6/7 levels than at other levels. In patients with a single-level procedure, the C5/6 or C6/7 was involved most, and the adjacent C6/7 or C5/6 was at high risk to degenerate.^[46]^ ASD was less common after multilevel surgery because these procedures usually included the higher-risk levels and had an end adjacent to segments that were at lower risk for the development of new disease. Our result also showed that anterior-approach subgroups had a higher prevalence of ASD than posterior-approach subgroups. This result could be due to the same reason as already mentioned, because the posterior approach usually was performed on patients with multilevel disc degeneration.

Several biomechanical studies suggested that fusion causes increased stress and strain on neighboring motion segments, which potentially contribute to accelerated degeneration, whereas TDR not only maintains physiologic motion at the operated level but also minimizes changes at the adjacent segments.^[89–91]^ In the clinical trials, ASD was also found in TDR patients after several years of follow-up.^[92]^ In our meta-analysis, the prevalence of reoperation ASD after TDR was 44% lower than after ACDF. The prevalence of radiographic ASD and symptomatic ASD was not calculated because the relatively small number of studies reported corresponding data. However, this result gives us some clues that TDR may have the advantage of decreasing the incidence of postoperative ASD.

As we know, the prevalence of ASD increased with the extension of follow-up time.^[11]^ This meta-analysis showed that with the addition of 1 follow-up year, the prevalence of radiographic ASD, symptomatic ASD, and reoperation ASD significantly increased by 2.79%, 1.43%, and 0.24%, respectively. Nevertheless, the cervical spine naturally undergoes degenerative changes with increasing age, and this fact poses a notable challenge when establishing ASD resulting from fusion^[93]^ versus that occurring simply as a natural aging process. Herkowitz et al^[94]^ studied patients with cervical radiculopathy after ACDF or posterior foraminotomy without fusion. After a mean of 4.2 follow-up years, 39% of patients developed ASD after fusion, but 50% of patients undergoing posterior foraminotomy also developed ASD at the operated and adjacent levels. Gore^[95]^ studied the natural history of cervical spondylotic disease in 159 asymptomatic patients and found that about 12% developed symptomatic spondylotic disease over a 10-year period. These studies imply that fusion is not the only factor that influences the risk of ASD, and future studies could provide more convincing evidence on this topic.

Several limitations should be considered when interpreting the findings of this study. First, there was a substantial amount of heterogeneity among the studies. Although potential sources of heterogeneity were explored by subgroup analyses of number of level, approach, and surgery types, none of them could sufficiently explain the heterogeneity. We did not conduct the subgroup analysis by age, gender, study design, or other factors because these data vary greatly. Second, not all of the included studies were designed for the prevalence study. Some of them did not provide detailed characteristics of patients with ASD, and this may have led to the imprecision of the pooled data. Third, the diagnosis criteria of ASD were not uniform among the included studies. Radiographic ASD, for example, could be better assessed and reported using classifications of severity. A multicentre prospective study using a single validated definition of ASD could provide a more accurate estimate of the prevalence of ASD after cervical surgery.

This meta-analysis provides detailed information on the prevalence of radiographic ASD, symptomatic ASD, and reoperation ASD after cervical surgery. This information should be useful to surgeons and patients to gain a better understanding of ASD during follow-up. However, because of the limitations noted earlier, the results of this meta-analysis should be interpreted with caution.
